# P09 A Phase 2, randomized, double-blind study to evaluate the safety, tolerability, and immunogenicity of a 21-valent pneumococcal conjugate vaccine (V116) in adults ≥50 years

**DOI:** 10.1093/jacamr/dlac133.013

**Published:** 2023-01-19

**Authors:** Tosin Omole, Jose Cardona, Neil J Fraser, Sandra Pagnussat, Richard Mularski, Charles Andrews, Nizar Daboul, Aditi Sapre, Jianing Li, Doreen Fernsler, Weifeng Xu, Nancy Gallagher, Lori Hall, Heather Platt

**Affiliations:** Merck & Co. Inc., Kenilworth, NJ 07033, USA; Indago Research & Health Center Inc., Hialeah, FL 33012, USA; Arcturus Healthcare PLC, Troy Internal Medicine Research Division, Troy, MI 48085, USA; QPS MRA LLC, Miami, FL 33143, USA; Kaiser Permanente Center for Health Research, Portland, OR 97227, USA; Diagnostics Research Group, San Antonio, TX 78229, USA; Advanced Medical Research, Maumee, OH 43537, USA; Merck & Co. Inc., Kenilworth, NJ 07033, USA; Merck & Co. Inc., Kenilworth, NJ 07033, USA; Merck & Co. Inc., Kenilworth, NJ 07033, USA; Merck & Co. Inc., Kenilworth, NJ 07033, USA; Merck & Co. Inc., Kenilworth, NJ 07033, USA; Merck & Co. Inc., Kenilworth, NJ 07033, USA; Merck & Co. Inc., Kenilworth, NJ 07033, USA

## Abstract

**Background:**

V116 is an investigational 21-valent PCV containing the following pneumococcal polysaccharides: 3, 6A, 7F, 8, 9N, 10A, 11A,12F, 15A, 16F, 17F, 19A, 20, 22F, 23A, 23B, 24F, 31, 33F, 35B, and a de-O-acetylated 15B (deOAc15B). This phase 2 study evaluated the safety, tolerability, and immunogenicity of V116 in pneumococcal vaccine-naïve adults compared with a 23-valent pneumococcal vaccine (PPSV23).

**Methods:**

Adults (*n*=508) ≥50 years were randomized 1:1 and received a single dose of V116 or PPSV23; randomization was stratified by age (50–64 years, 65–74 years, and ≥75 years). Adverse events (AEs) were collected following vaccination. Pneumococcal serotype-specific opsonophagocytic activity (OPA) and IgG were measured before and 30 days after vaccination (Day 30). Primary immunogenicity objectives were to assess the noninferiority of V116 to PPSV23 for the shared serotypes and the superiority of V116 to PPSV23 for the unique serotypes.

**Results:**

There were no vaccine-related serious AEs or vaccine-related deaths. V116 met noninferiority criteria compared with PPSV23 for all shared serotypes [based on the lower bound of the 95% CIs for the estimated OPA GMT ratios (V116/PPSV23) being >0.33 at Day 30] and met superiority criteria for the unique serotypes [based on the lower bound of the 95% CIs for the estimated OPA GMT ratios (V116/PPSV23) being >1.0 at Day 30].

**Conclusions:**

In adults ≥50 years, V116 is well tolerated with a safety profile comparable to PPSV23, elicits an immune response that is noninferior to PPSV23 for the shared serotypes and superior to PPSV23 for the unique serotypes.

